# Oxidative Stress in Cancer-Prone Genetic Diseases in Pediatric Age: The Role of Mitochondrial Dysfunction

**DOI:** 10.1155/2016/4782426

**Published:** 2016-04-27

**Authors:** Serafina Perrone, Federica Lotti, Ursula Geronzi, Elisa Guidoni, Mariangela Longini, Giuseppe Buonocore

**Affiliations:** ^1^Department of Molecular and Developmental Medicine, University of Siena, 53100 Siena, Italy; ^2^UOC Patologia Clinica, AOUS Siena, 53100 Siena, Italy

## Abstract

Oxidative stress is a distinctive sign in several genetic disorders characterized by cancer predisposition, such as Ataxia-Telangiectasia, Fanconi Anemia, Down syndrome, progeroid syndromes, Beckwith-Wiedemann syndrome, and Costello syndrome. Recent literature unveiled new molecular mechanisms linking oxidative stress to the pathogenesis of these conditions, with particular regard to mitochondrial dysfunction. Since mitochondria are one of the major sites of ROS production as well as one of the major targets of their action, this dysfunction is thought to be the cause of the prooxidant status. Deeper insight of the pathogenesis of the syndromes raises the possibility to identify new possible therapeutic targets. In particular, the use of mitochondrial-targeted agents seems to be an appropriate clinical strategy in order to improve the quality of life and the life span of the patients.

## 1. Introduction

Reactive oxygen species (ROS) have crucial roles in many physiological and pathophysiological processes. A delicate balance between oxidants and antioxidants is essential for physiological functioning. On the contrary, the loss of this balance usually leads to dysfunctions and cellular damage at various levels, including membrane phospholipids, proteins, and nucleic acids [1–6].

In 1956 Harman postulated the free radical theory of ageing, according to which a redox imbalance and a ROS surplus are involved in the cellular damage that accompanies ageing and age-related diseases such as neurodegenerative diseases and cancer [7]. Since then, a huge body of literature has been produced on the role of oxidative stress (OS) in ageing and carcinogenesis, and a clear link between OS and the development of specific types of cancer has been ascertained [8–11]. In particular, the DNA damage inflicted by ROS contributes to the initiation and progression of carcinogenesis. ROS are able to react with DNA, damaging nitrogenous bases or producing double-strand breaks. They can also oxidize lipids and proteins, resulting in the production of intermediate species which in turn react with DNA. Several repair mechanisms intervene in removing DNA injuries; however, disrepair of DNA damage may occur in some cases, resulting in base substitutions or deletions leading to cancer development. In addition, DNA repair mechanisms have the tendency to decay with age: this leads to progressive accumulation of DNA injuries that accounts for the increased incidence of cancer with age [3, 12–15].

A second theory proposed to explain the mechanisms involved in ageing and in age-related diseases, including cancer, is the mitochondrial theory of ageing, postulated in 1984 by Miquel and Fleming and based on the presence of a mitochondrial dysfunction [16]. Increased ROS production, accumulation of damaged mitochondrial DNA (mtDNA), and progressive respiratory chain dysfunction are the three main principles of the theory. With age, a vicious cycle takes place: increased ROS production causes accumulation of oxidative damage in mtDNA, which is more sensitive to ROS-induced damage than nuclear DNA; mutated mtDNA codifies malfunctioning subunits of respiratory complexes that in turn increase ROS production [17–20]. Signs of altered mitochondrial activity can be recognized in many OS related disorders, thus proving the existence of a strict connection between OS and mitochondrial dysfunction [21].

OS is a hallmark in several genetic diseases. In particular, evidence has been reported of an OS intervention in the pathogenesis of a number of cancer-prone genetic syndromes. In some of these diseases a mitochondrial dysfunction has also been demonstrated [22].

Taking into account the link between OS and carcinogenesis and the pivotal role exerted by mitochondrial dysfunction, the use of mitochondrial-targeted antioxidants and micronutrients might be a good clinical strategy to prevent cancer development in these syndromes.

## 2. Mitochondrial Dysfunction and Cancer Development: Mitochondrial-Targeted Antioxidants

Abnormalities in mitochondrial functions have been reported in several human pathologies, including cardiologic, haematologic, autoimmune, neurologic, and psychiatric disorders. One of the main lines of research in this respect investigates the link between mitochondrial dysfunction and cancer [21]. In cancer cells the increased ROS production is linked to mtDNA mutations and to alterations of the bioenergetics and the biosynthetic state of cancer cells [23]. Cancer cells show indeed several metabolic alterations, including increased fatty acid synthesis and glutamine metabolism, and an increased aerobic glycolysis [24, 25]; the latter feature is known as the “Warburg effect” and is thought to be due to defective mitochondria [26]. The switch towards aerobic glycolysis enables cancer cells to use glucose supplies for the biosynthesis of macromolecules, to support their rapid growth. ROS surplus can also determine the peroxidation of fatty acids in mitochondrial membranes: for example, the peroxidation of mitochondrial phospholipid cardiolipin leads to the formation of reactive aldehydes which in turn react with proteins and DNA [23]. Alterations of mitochondrial proteins are involved in mitochondrial dysfunctions characteristic of cancer cells. Moreover, dysfunctional mitochondria are able to modulate cell cycle, gene expression, metabolism, and cell viability [27].

In view of these findings, a supportive therapeutic approach based on the use of mitochondrial-targeted substances might be an appropriate strategy. A mitochondrial nutrient is an agent able to protect mitochondria from oxidative damage and to improve mitochondrial function by preventing generation of ROS, scavenging free radicals, and preventing oxidized inactive proteins accumulation. It can also repair oxidative damage by enhancing antioxidant defense systems [28–30]. A number of mitochondrial cofactors have been tested in several clinical trials to verify their potential benefits. Among them, the most studied are alpha-lipoic acid (ALA), coenzyme Q_10_ (CoQ_10_), and L-carnitine. ALA is a dithiol compound, derived from octanoic acid, that is known as an essential cofactor for mitochondrial bioenergetics' enzymes. It is a natural antioxidant found in every cell of the body and it is able to trigger the mitochondrial pathway of apoptosis in cancer cells [31, 32]. CoQ_10_ is an endogenous lipid synthesized by the human organism and also introduced in small amounts through the diet. It is an electron acceptor and donor, and it may occur in an oxidized form (ubiquinone) and a reduced form (ubiquinol). It is important for the maintenance of mitochondrial homeostasis and the prevention of free radical production; in the form of ubiquinol, it also acts directly as a scavenger [33–35]. L-Carnitine (*β*-hydroxy-*γ*-trimethyl-ammonium butyric acid) is involved in mammalian lipid metabolism: it is required in the transport of activated fatty acids from the cytosol into the mitochondrial matrix, where *β*-oxidation takes place. In addition, it seems to take part in the repair of induced single-strand DNA breaks and in the protection of DNA from ROS [36–38].

## 3. Oxidative Stress and Mitochondrial Dysfunction in Cancer-Prone Genetic Diseases

A group of genetic diseases, including Down syndrome (DS), Ataxia-Telangiectasia (AT), Fanconi Anemia (FA), Bloom syndrome (BS), and Werner syndrome (WS), show OS and mitochondrial dysfunction as a phenotypic hallmark. These genetic disorders share, among other things, predisposition to cancer development and premature ageing.

AT is characterized by progressive neurodegeneration, immunodeficiency, oculocutaneous telangiectasias, endocrine abnormalities, high cancer incidence, genome instability, and hypersensitivity to ionizing radiation [39, 40]. The lifetime prevalence of cancer is about 40% [41]. In children with AT the most frequent cancer cases are acute lymphocytic leukemia and lymphoma [42]. AT is an autosomal recessive disorder caused by mutational inactivation of* ATM* gene, located on the long arm of chromosome 11.* ATM* gene encodes a protein belonging to the PI3/PI4-kinase family. The ATM protein is an important cell cycle checkpoint kinase involved in the repair response to DNA double-strand breaks [43, 44]. Loss of ATM function leads to genomic instability with chromosome breaks, translocations, and aneuploidy [45]. A link between OS and AT has been demonstrated in several studies [46–49]; recent research has provided some possible new mechanisms for oxidative damage associated with ATM deficiency that are independent of the DNA damage response pathway. In particular, ATM seems to be able to influence ROS production through the modulation of mitochondrial activity [50–52]. AT cells established from AT patients show an abnormal structural organization of mitochondria with a decreased membrane potential and an increased basal expression level of several nuclear DNA-encoded genes whose proteins are involved in oxidative damage response and are targeted to mitochondria. In addition, they show decreased overall mitochondrial respiratory activity: this activity could be rescued by treating the cells either with ALA or by the expression of full-length ATM, suggesting that the protein is required for the regulation of mitochondrial dysfunction [53]. In the light of these data, the use of antioxidants directed at mitochondrial ROS could be a therapeutic strategy for AT patients. D'Souza et al. demonstrated that reducing mitochondrial ROS through overexpression of catalase targeted to mitochondria (mCAT) alleviates AT-related pathology in* ATM*-deficient mice, with particular regard to cancer pathology [54]. Berni et al. studied the effect of pretreatment with L-carnitine on DNA damage in normal and ATM-deficient cells established by AT patients and found that L-carnitine enhanced the rate and extent of DNA repair in AT cell lines; a reduction of all types of chromosomal aberrations was also observed [55].

FA is characterized by bone marrow failure leading to pancytopenia, physical abnormalities (including short stature, abnormal skin pigmentation, malformation of the thumbs, forearms, skeletal system, eyes, kidneys and urinary tract, ears, heart, gastrointestinal system, and central nervous system), type 2 diabetes mellitus, hypogonadism, and developmental delay [56]. Affected patients have an increased risk of malignancy, primarily acute myeloid leukemia. The risk of solid tumors is also increased [57]. FA is caused by mutation in 15 known genes whose functions are especially linked to DNA repair pathways [58–61]. There is a huge body of literature on the link between OS and FA, with numerous studies since the 1970s. The cells from FA patients show a prooxidant state and some of the genes linked to the syndrome's pathogenesis encode proteins involved in redox homeostasis. Moreover, FA patients display downregulation of major antioxidant defense genes [62–65]. The presence of mitochondrial defects in FA cells has been highlighted by recent literature: these defects seem to be directly connected to the increased ROS production and to the concurrent depletion of antioxidant defenses. In particular, FA cells show excess formation of mitochondrial ROS with a decreased mitochondrial membrane potential, decreased ATP production, impaired oxygen uptake, abnormalities in mitochondrial ultrastructure, and inactivation of mitochondrial activities involved in bioenergetics pathways and ROS detoxification [66–68]. Ponte et al. studied the protective effect of ALA and N-acetylcysteine (NAC) on chromosome instability in cells established from FA patients and found that the micronutrients cocktail is able to improve the genetic stability of FA lymphocytes* in vitro* [68]. A possible role for mitochondrial nutrients as chemopreventive agents in FA is suggested by these data. In this regard, a pilot study on the use of quercetin in children with FA has been set up by the Cincinnati Children's Hospital Medical Center and is currently recruiting participants. Primary outcome of the study is to assess the feasibility, toxicity, and pharmacokinetics of oral quercetin therapy in FA; secondary outcomes include assessment of the impact of quercetin on ROS reduction (Clinical Trial Identifier: NCT01720147). Quercetin (3,3′,4′,5,7-pentahydroxyflavone) is a flavonoid with anti-inflammatory and antioxidant properties and seems also to enhance mitochondrial functionality [69, 70].

DS is one of the most common genetic anomalies. The main features of the syndrome are cognitive impairment, craniofacial dysmorphism, gastrointestinal abnormalities, congenital heart defects, endocrine abnormalities, neuropathology leading to dementia, and immunological defects. In approximately 95% of patients, DS is caused by full trisomy 21; the remaining cases are linked to mosaicism and translocations [71]. Affected children show a higher incidence of leukemia than the general population [72, 73]. DS seems to be characterized by a chronic prooxidant state: ongoing OS can be demonstrated from embryonic life and evidence of mitochondrial dysfunction has been reported, including alteration in the membrane potential, oxidative damage to mtDNA, ultrastructure changes such as abnormally shaped mitochondria, and diminished levels of microtubules [22, 74, 75]. Chromosome 21 contains several genes implicated in OS, above all Cu/Zn superoxide dismutase (*SOD1*). SOD1 is implicated in antioxidant defense: it catalyzes the dismutation of ^∙^O_2_^−^ to molecular oxygen (O_2_) and H_2_O_2_, which can be converted by catalase and glutathione peroxidase to water. The triplication of chromosome 21 leads to an imbalance in the ratio of SOD1 to catalase and glutathione peroxidase, resulting in the accumulation of H_2_O_2_ [76, 77]. Tiano et al. evaluated the effect of CoQ_10_ administration to DS patients. At the beginning a mild protective effect on DNA was demonstrated at the cellular level, but the treatment failed to modify the overall oxidative damage at the patient level. After a longer follow-up and prolonged treatment, an age-specific reduction in the percentage of cells showing the highest amount of oxidized bases was highlighted, indicating a potential role of CoQ_10_ in modulating DNA repair mechanisms [78, 79].

BS is a rare, autosomal recessive disorder exhibiting numerous clinical features including sensitivity to sunlight, growth retardation, immunological disorders, and predisposition to cancer [80, 81]. Cells established by BS patients show excess DNA damage with a decreased glutathione disulfate : glutathione (GSSG : GSH) ratio [21, 82].

Finally, progeroid syndromes are a group of disorders characterized by clinical features mimicking physiological ageing at an early age. Several causative genes have been identified: genes encoding DNA repair factors (DNA helicases) and genes affecting the structure or posttranslational maturation of lamin A, which is a major nuclear component. Moreover, several animal models show abnormal mitochondrial function [83]. In particular, in WS involvements of the defective WRN protein in DNA stability and in redox balance have been observed and mitochondrial ultrastructure anomalies were found in cells from WS mouse model [21, 84].

## 4. Oxidative Stress and Beckwith-Wiedemann, Costello, and Prader-Willi Syndromes

BWS is a genomic imprinting disorder characterized by abdominal wall defects, macroglossia, pre- and postnatal overgrowth, neonatal hypoglycemia, visceromegaly, and increased risk of developing cancer in childhood, such as Wilms' cancer, hepatoblastoma, neuroblastoma, adrenocortical carcinoma, and rhabdomyosarcoma. The lifetime risk of developing cancer is approximately 7.5% [85]. The syndrome is associated with alterations in 2 distinct imprinting domains on 11p15: a telomeric domain containing the* H19* and* IGF2* genes and a centromeric domain including the* KCNQ1OT1* and* CDKNIC* genes. Disorders of imprinting in the telomeric domain are associated with overgrowth and tumor development; imprinting defects at* KCNQ1OT1* are associated with the development of other embryonal tumors [85].

Costello syndrome is a rare genetic disease characterized by coarse facies, short stature, loose folds of skin on the hands and feet, severe feeding difficulties and failure to thrive, cardiac anomalies, developmental disability, and increased risk of malignancies, especially rhabdomyosarcoma, with an approximately 15% lifetime risk. The only gene currently known to be associated with the syndrome is Harvey rat sarcoma viral oncogene homolog (*HRAS*). Defects in this gene are implicated in a variety of cancers, including bladder cancer, follicular thyroid cancer, and oral squamous cell carcinoma [86]. The protein encoded by* HRAS* belongs to the Ras-mitogen-activated protein kinase (MAPK) pathway. Recent data demonstrated a functional connection between the Ras-MAPK pathway and mitochondrial function, and functional defects in mitochondrial respiration could be induced by oncogenic HRAS transformation, suggesting a possible role for mitochondrial dysfunction in the pathogenesis of CS [87–89].

By measuring a redox biomarker profile, the presence of a prooxidant state in patients affected by CS and BWS was documented. The administration of potassium ascorbate with ribose (PAR), which acts as antioxidant, determined a progressive decrease in OS biomarkers until their normalization, together with an improvement in the clinical conditions of the patients. No neoplastic disease was observed during a follow-up period of 10 years [90].

Potassium ascorbate is a salt derived from natural ascorbic acid; it is totally nontoxic and has antioxidant effects, combining the antioxidant action of vitamin C with the stabilizing intracellular effects of potassium. The ribose acts as a catalyst strengthening the action of potassium ascorbate [91, 92]. Ascorbic acid has been used in the prevention of cancer with promising results [93]. Mitochondria may be one of the principal targets of its activity: at higher concentrations vitamin C seems to increase ATP production by increasing mitochondrial electron flux and to induce apoptosis in cancer cells [94].

PAR supplementation gave promising results also in Prader-Willi syndrome (PWS), a genomic imprinting disorder whose most important feature is severe obesity leading to atherosclerosis and type 2 diabetes mellitus, in which a close relationship with OS has been widely demonstrated [95]. Interestingly, mitochondrial dysfunction was found in an imprinting center deletion mouse model of PWS, suggesting that an altered mitochondrial activity may contribute to the PWS pathogenesis [96]. Prader-Willi syndrome is not a cancer-prone disease; however, in recent years cases of early-onset cancer have been reported in PWS patients, probably due to the increased life expectancy, raising the question whether PWS predisposes to cancer development [97]. Indeed, there is evidence of a potential role of genomic imprinting and DNA methylation in human cancer [98, 99]. In addition,* Necdin* gene, which maps to chromosome 15q11–13, the region implicated in the pathogenesis of PWS, may have a potential tumor suppressor role, and it seems to be downregulated and hypermethylated or mutated in cancer [100, 101].

Antioxidant supplementation (PAR) in a PWS patient was associated with a progressive reduction of OS biomarkers occurring together with improvement in the clinical aspects of the patient, including the lack of development of the characteristic obesity [95]. Studies on relationship between oxidative stress and BWS, CS, and PWS, although being preliminary and based on a small group of patients, raise the prospect of future clinical trials based on larger case histories and with longer follow-up periods.

## 5. Conclusions

Oxidative stress is an important hallmark in several genetic diseases characterized by predisposition to tumor development and/or premature ageing. Studying the molecular mechanisms linking OS to the pathogenesis of these conditions allows identifying new possible therapeutic targets. Antioxidants administration to the affected patients might counteract their prooxidant state. Since a prooxidant state is often associated with mitochondrial dysfunction, the use of mitochondrial-targeted agents might be an appropriate clinical strategy in order to improve the quality of life and the life span of the patients.
